# Differential Regulation of the Excitability of Prefrontal Cortical Fast-Spiking Interneurons and Pyramidal Neurons by Serotonin and Fluoxetine

**DOI:** 10.1371/journal.pone.0016970

**Published:** 2011-02-24

**Authors:** Ping Zhong, Zhen Yan

**Affiliations:** Department of Physiology and Biophysics, School of Medicine and Biomedical Sciences, State University of New York at Buffalo, Buffalo, New York, United States of America; Neuroscience Campus Amsterdam, VU University, The Netherlands

## Abstract

Serotonin exerts a powerful influence on neuronal excitability. In this study, we investigated the effects of serotonin on different neuronal populations in prefrontal cortex (PFC), a major area controlling emotion and cognition. Using whole-cell recordings in PFC slices, we found that bath application of 5-HT dose-dependently increased the firing of FS (fast spiking) interneurons, and decreased the firing of pyramidal neurons. The enhancing effect of 5-HT in FS interneurons was mediated by 5-HT_2_ receptors, while the reducing effect of 5-HT in pyramidal neurons was mediated by 5-HT_1_ receptors. Fluoxetine, the selective serotonin reuptake inhibitor, also induced a concentration-dependent increase in the excitability of FS interneurons, but had little effect on pyramidal neurons. In rats with chronic fluoxetine treatment, the excitability of FS interneurons was significantly increased, while pyramidal neurons remained unchanged. Fluoxetine injection largely occluded the enhancing effect of 5-HT in FS interneurons, but did not alter the reducing effect of 5-HT in pyramidal neurons. These data suggest that the excitability of PFC interneurons and pyramidal neurons is regulated by exogenous 5-HT in an opposing manner, and FS interneurons are the major target of Fluoxetine. It provides a framework for understanding the action of 5-HT and antidepressants in altering PFC network activity.

## Introduction

The prefrontal cortex (PFC) is a central brain region controlling high-level executive functions and goal-directed behaviors [1]. Clinical, neuropsychological, and imaging studies have indicated that several neuropsychiatric disorders, including depression, anxiety and schizophrenia, are related to the deficits in cognitive and emotional processes subserved by PFC [2]–[5]. PFC receives a dense serotonergic innervation from the dorsal and median raphe nuclei [6]. Growing evidence suggests that the serotonergic system plays an important role in regulating prefrontal functions [7]–[11]. The serotonin system is also heavily involved in depressive disorders [12]–[14], and fluoxetine, which enhances serotonin levels by blocking its reuptake, has been the most successful antidepressant drug [15].

The cellular mechanism underlying the actions of 5-HT and fluoxetine in PFC has been largely unknown. PFC activity is control by the excitability of two major neuronal populations: glutamatergic excitatory pyramidal neurons and GABAergic inhibitory interneurons [16]. The parvalbumin-expressing fast-spiking (FS) interneuron network generates gamma oscillations [17], [18], which is critical for cognitive tasks such as attention and sensory processing [19], [20]. Specific deficits in PFC FS interneurons have been found in schizophrenia patients [21]. Moreover, alterations of prefrontal cortical activity are considered as an important causal factor for major depression [22], which provides a basis for the treatment of depression with brain stimulation [23].

Both PFC principal neurons and interneurons contain multiple 5-HT receptors, with a particular abundance of the 5-HT_1A_ and 5-HT_2A_ subtypes [24]–[26]. Blockade of PFC 5-HT_2A_ receptors has been found to impair working memory, which involves actions at both excitatory and inhibitory elements within PFC circuitry [27]. Despite the findings on the effect of 5-HT on glutamatergic and GABAergic synaptic responses in PFC pyramidal neurons [28]–[32], it remains unclear about the impact of 5-HT or fluoxetine on the intrinsic excitability of PFC interneurons and pyramid neurons.

In this study, we have found that 5-HT produces opposing effects on the action potential firing of PFC FS interneurons and pyramidal neurons. Fluoxetine treatment *in vitro* (acute) or *in vivo* (chronic) mainly alters the intrinsic excitability of FS interneurons, but not pyramidal neurons. These results provide a framework for understanding the action of 5-HT and antidepressants in altering PFC network activity.

## Results

### The effect of serotonin on the excitability of FS interneurons and pyramidal neurons in PFC

**Figure 1:**
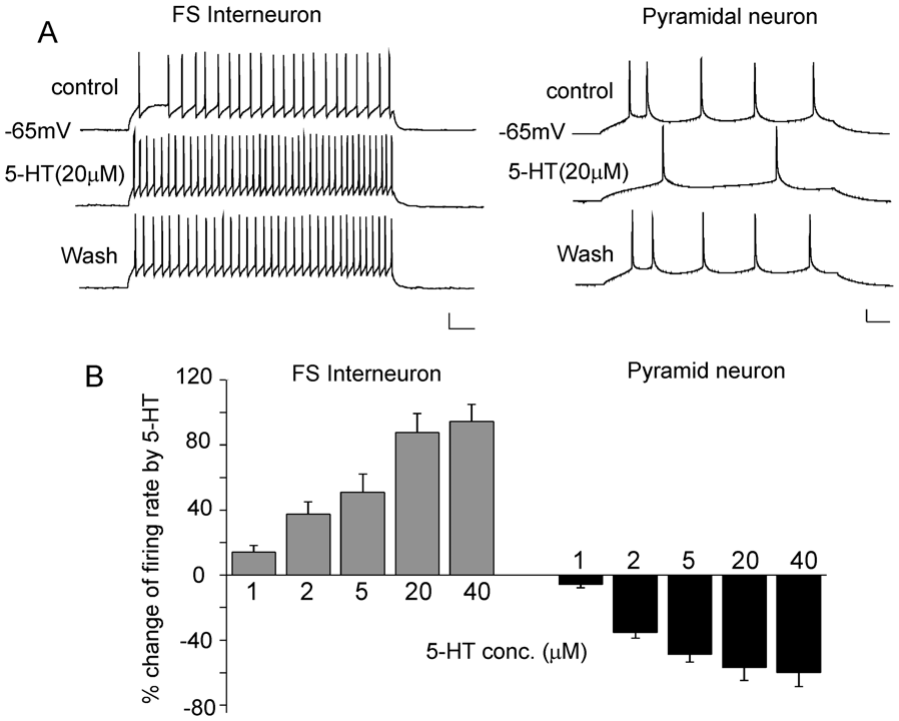
The effect of 5-HT on AP firing in FS interneurons and pyramidal neurons of PFC. A, Representative AP recordings showing the effect of 5-HT (20 µM) in a FS interneuron and a pyramidal neuron. Scale bars: 20 mV, 50 ms. B, Cumulative data (mean ± SEM) showing the percentage change of the firing rate by different doses of 5-HT in FS interneurons and pyramidal neurons.

To understand the effect of serotonin on the excitability of cortical neuronal populations, we conducted whole-cell current-clamp recordings to examine the action potential (AP) firing in FS interneurons and pyramidal neurons located at layer 3–5 of PFC from young adult rats. Pyramidal neurons were identified by their triangular soma and a clear apical dendrite, whereas interneurons were characterized by a round or oval cell body and the lack of a visible apical dendrite under infrared video microscopy. Action potentials were elicited by injecting a depolarizing current pulse. FS interneurons generated trains of spikes of short durations (base duration: ∼2 ms) followed by a strong fast afterhyperpolarization (fAHP) and were characterized by their fast spikes discharged at high frequencies with little frequency adaptation (Fig. 1A) [33], [34]. In contrast, pyramidal neurons fired long-duration (base duration: ∼4.5 ms) and low frequency spikes that showed adaptation followed by a weak fAHP (Fig. 1A).

Bath application of 5-HT (20 µM) significantly increased the firing rate in FS interneurons, while decreased the firing rate in pyramidal neurons (Fig. 1A). Both the enhancing and the reducing effects were concentration-dependent (Fig. 1B, interneurons: 1 µM, 16.2±3.9%, 2 µM, 37.4±7.6%, 5 µM, 50.8±11.3%, 20 µM, 87.6±11.7%, 40 µM, 94.5±10.6%, n = 5–10 for each dose; pyramidal neurons: 1 µM, −5.7±2.1%, 2 µM, −35.2±3.6%, 5 µM, −48.6±4.8%, 20 µM, −56.8±7.9%, 40 µM, −59.9±8.7%, n = 5–7 for each dose). To check whether synaptic activity influences the effect of 5-HT on APs, we used the AMPAR antagonist CNQX (20 µM), NMDAR antagonist APV (50 µM) and GABA_A_R antagonist bicuculline (10 µM) to block excitatory and inhibitory neurotransmission. 5-HT (20 µM) caused a similar enhancement of the firing rate in FS interneurons in the presence of these antagonists (84.7±13.3%, n = 4), suggesting that 5-HT may change the neuronal excitability by altering their intrinsic properties.

### Different 5-HT receptors mediate the distinct effects of 5-HT in FS interneurons and pyramidal neurons

**Figure 2:**
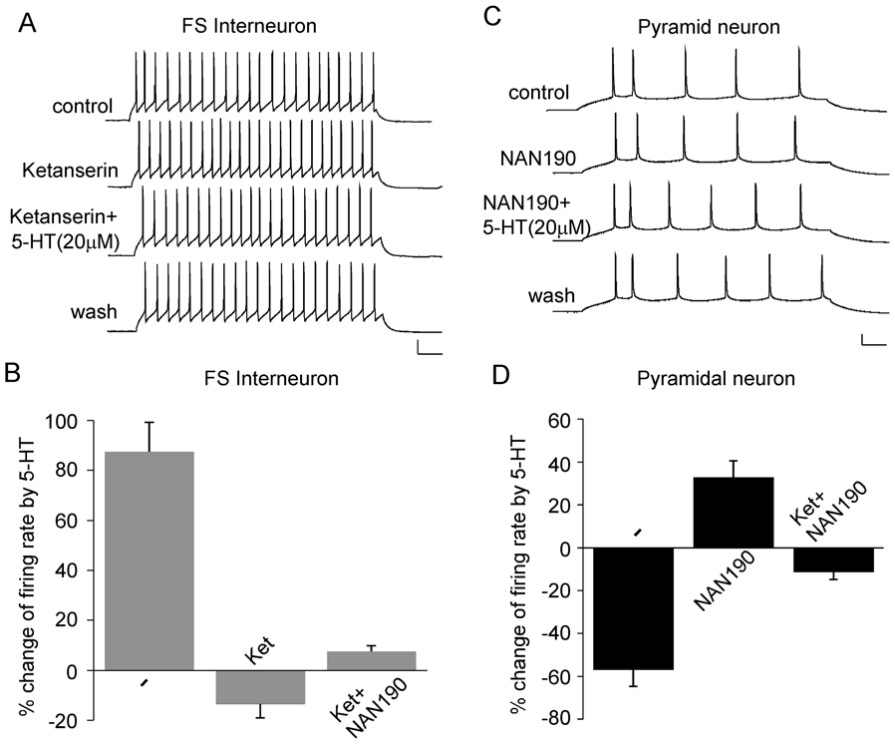
Different 5-HT receptors mediate the effect of 5-HT on AP firing in PFC FS interneurons and pyramidal neurons. A, C, Representative AP recordings showing the effect of 5-HT (20 µM) in the presence of the 5-HT_2_ antagonist Ketanserin (10 µM) or the 5-HT_1_ antagonist NAN190 (10 µM) in a FS interneuron and a pyramidal neuron. Scale bars: 20 mV, 50 ms. B, D, Cumulative data (mean ± SEM) showing the percentage changes of the firing rate by 5-HT (20 µM) in the presence of different antagonists in FS interneurons and pyramidal neurons.

Serotonin can have both inhibitory and excitatory functions in neuronal networks through the activation of different 5-HT receptors [35]. We next examined which 5-HT receptors mediate the effects of 5-HT on APs in FS interneurons or pyramidal neurons. As shown in Fig. 2A and 2B, in FS interneurons, the specific 5-HT_2_ antagonist Ketanserin (10 µM) turned the enhancing effect of 5-HT to a small reduction (−13.3±5.7%, n = 5), and Ketanserin itself had little effect on APs. On the other hand, in pyramidal neurons (Fig. 2C and 2D), the specific 5-HT_1_ antagonist NAN190 (10 µM) turned the reducing effect of 5-HT to a small enhancement (32.8±7.6%, n = 7), and NAN190 itself did not alter APs. Blocking both 5-HT_1_ and 5-HT_2_ receptors with Ketanserin and NAN190 largely eliminated 5-HT effects (interneuron: 7.6±2.3%, n = 4; pyramidal neuron: −11.2±3.6%, n = 4). These data suggested that the enhancing effect of 5-HT in FS interneurons is predominantly mediated by 5-HT_2_ receptors, while the reducing effect of 5-HT in pyramidal neurons is mainly mediated by 5-HT_1_ receptors.

### The effect of *in vitro* or *in vivo* fluoxetine administration on the excitability of FS interneurons and pyramidal neurons in PFC

**Figure 3:**
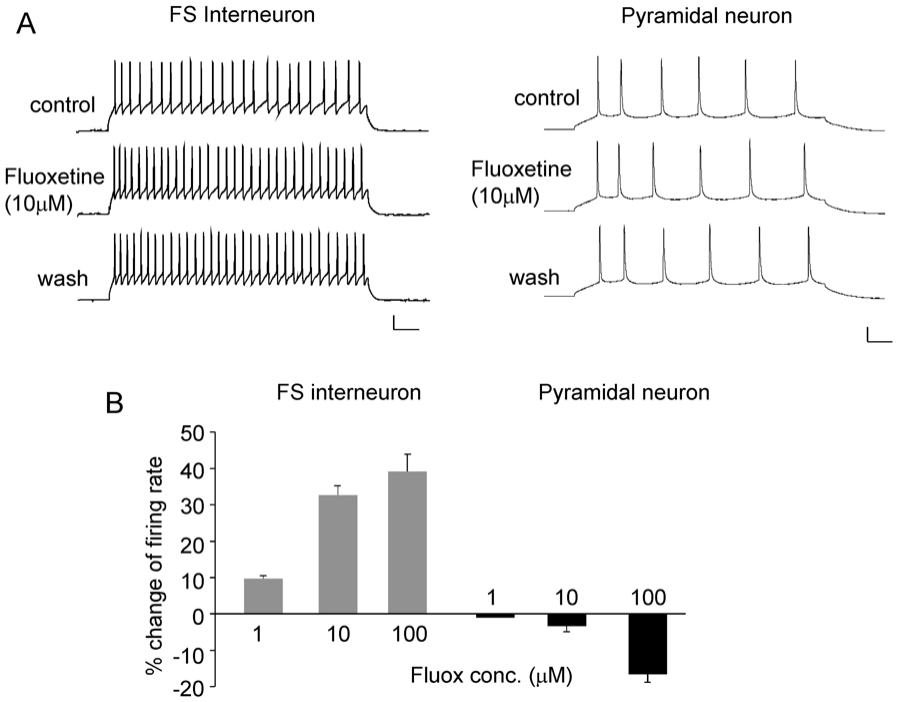
The effect of *in vitro* fluoxetine application on AP firing in FS interneurons and pyramidal neurons of PFC. A, Representative AP recordings showing that effect of bath application of fluoxetine (10 µM) in a FS interneuron and a pyramidal neuron. Scale bars: 20 mV, 50 ms. B, Cumulative data (mean ± SEM) showing the percentage change of the firing rate by different doses of fluoxetine in FS interneurons and pyramidal neurons.

Fluoxetine, a selective serotonin reuptake inhibitor, is the most widely used antidepressant drug [36]. Next, we examined whether endogenous activation of 5-HT receptors by fluoxetine could also alter the excitability of PFC neurons. As shown in Fig. 3A, bath application of fluoxetine (10 µM) significantly increased the firing rate of FS interneurons, but had little effect on pyramidal neurons. A higher dose of fluoxetine (100 µM) gave similar enhancement in FS interneurons (Fig. 3B, 10 µM: 32.7±2.3%, 100 µM: 39.2±4.8%, n = 5), and only slightly decreased the firing rate of pyramidal neurons (Fig. 3B, 10 µM: −3.3±1.4%, 100 µM: −16.5±2.2%, n = 5–6). These data suggest that FS interneurons are more sensitive to the *in vitro* application of fluoxetine.

**Figure 4:**
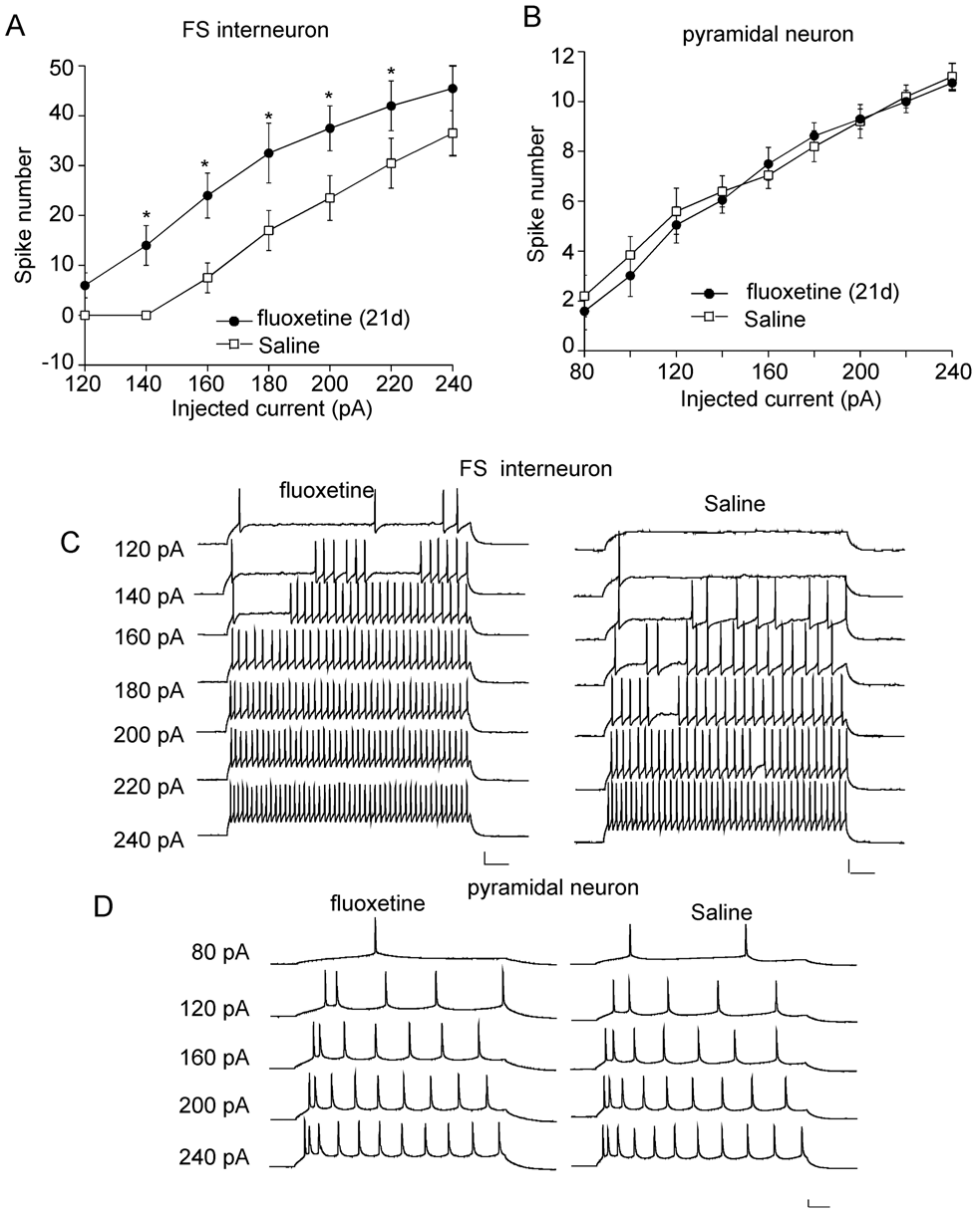
The effect of long-term *in vivo* fluoxetine treatment on the excitability of FS interneurons and pyramidal neurons. A, B, Plot of spike numbers (mean ± SEM) in response to different current (500 ms) injections in PFC FS interneurons and pyramidal neurons from rats i.p. injected with saline or fluoxetine for 21 days. *: p<0.01, *t* test. C, D, Representative AP recordings in response to injected currents in FS interneurons and pyramidal neurons from saline- or fluoxetine-injected rats. Scale bars: 20 mV, 50 ms.

Since the therapeutic effects of fluoxetine are not attained in patients until 2–3 weeks after the beginning of treatment [37], the 21-day fluoxetine administration regimen has been widely used as an effective way for chronic antidepressant testing [38], [39]. This regimen can cause behavioral, biochemical and physiological changes that are associated with anti-depression efficacy of fluoxetine [40], [41]. So we injected rats with fluoxetine (10 mg/kg/day) for 21 days to examine the impact of long-term fluoxetine treatment on the excitability of PFC neurons. Saline injections were used a control. As shown in Fig. 4, the intrinsic excitability, as measured by the number of spikes elicited by injected depolarizing current pulses (120–240 pA, 500 ms), was significantly increased in PFC FS interneurons from fluoxetine-injected rats (160 pA: Saline: 7.5±3, Fluox: 24±4.5; 180 pA: Saline: 17±4, Fluox: 32.5±6; 200 pA: Saline: 23.5±4.5, Fluox: 37.5±4.5, n = 5 for each group), while the excitability of PFC pyramidal neurons was unchanged by fluoxetine injection (120 pA: Saline: 5.6±0.9, Fluox: 5.1±0.7; 160 pA: Saline: 7.1±0.52, Fluox: 7.5±0.7; 200 pA: Saline: 9.2±0.7, Fluox: 9.3±0.4, n = 6 for each group). These results suggest that long-term fluoxetine treatment mainly increased the excitability of FS interneurons, which could lead to the enhanced inhibitory circuit in PFC.

### The effect of serotonin on the excitability of PFC neurons in animals with long-term fluoxetine treatment

**Figure 5:**
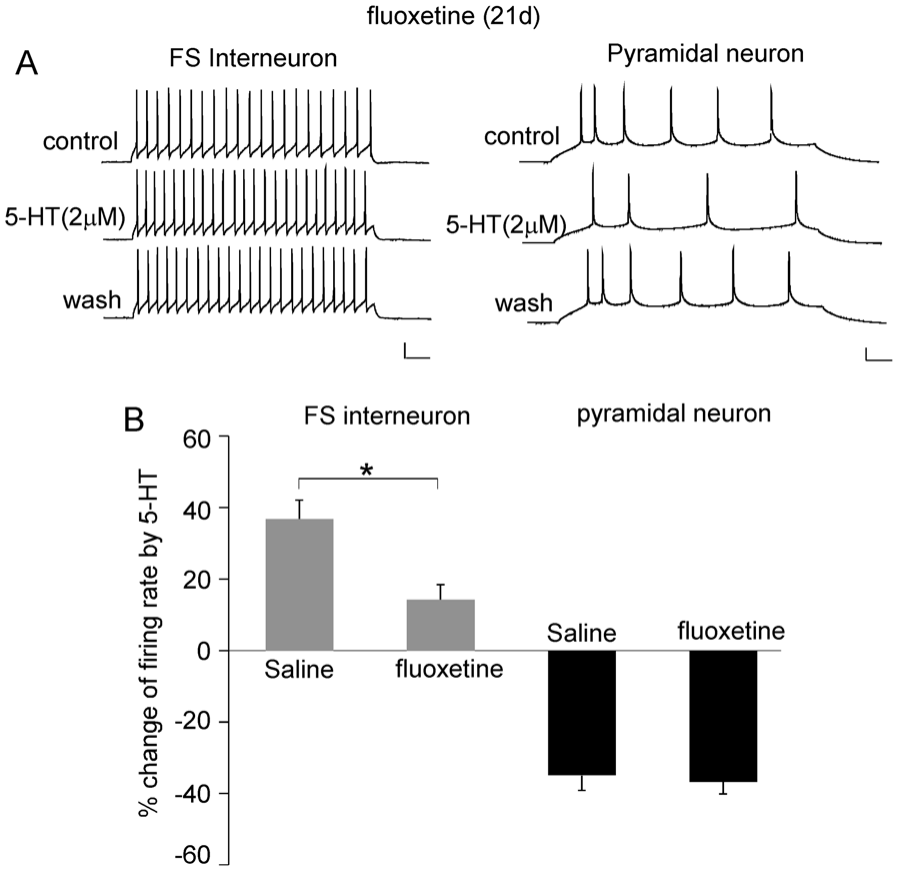
The effect of 5-HT on AP firing in PFC neurons from fluoxetine-treated rats. A, Representative AP recordings showing the effect of 5-HT (2 µM) in a FS interneurons and a pyramidal neuron from saline- or fluoxetine-injected rats. Scale bars: 20 mV, 50 ms. B, Cumulative data (mean ± SEM) showing the percentage change of the firing rate by 5-HT (2 µM) in FS interneurons and pyramidal neurons from saline- or fluoxetine-injected rats. *: p<0.01, *t* test.

Next, we examined whether exogenous application of 5-HT had any effect on PFC in the rats with 20-day fluoxetine injection. As shown in Fig. 5, the enhancing effect of 5-HT (2 µM) on FS interneuron APs was significantly attenuated in fluoxetine-injected rats, compared to saline-injected rats (saline: 36.8±5.3%, n = 6, fluox: 14.3±4.2%, n = 5, p<0.01, *t* test). In contrast, the reducing effect of 5-HT (2 µM) on pyramidal neuron APs was not altered (saline: −35.2±3.6%, n = 6, fluox: −36.7±3.3%, n = 6). It suggests that chronic fluoxetine treatment significantly occluded the effect of 5-HT in FS interneurons, while the excitability of pyramidal neurons is regulated by 5-HT via a fluoxetine-independent mechanism.

## Discussion

In this study, we have revealed the effect of serotonin and fluoxetine on the intrinsic excitability of PFC FS interneurons and pyramidal neurons. Since action potential firing is the final output of neurons, our results provide a framework for understanding the role of serotonin and fluoxetine in regulating PFC network activity, which is crucial for PFC-mediated cognitive processes such as working memory [42]. It is known that FS interneurons play a central role in determining the timing and spatial selectivity of pyramidal firing [43], thus shaping the physiological outcome of the PFC network. By increasing the excitability of PFC FS interneurons, the feedforward inhibition could be enhanced by serotonin and fluoxetine. The decreased excitability of PFC pyramidal neurons in response to serotonin could further dampen the circuit activity.

5-HT exerts complex effects on central neurons, depending on the cell type, the channel target, the expression of 5-HT receptor subtypes and the developmental stage [35], [44], [45]. 5-HT_1A_ and 5-HT_2A_ receptors have been found in PFC pyramidal neurons and interneurons [25], while it is unclear which receptor plays the dominant role in these different types of neurons. Bath administration of 5-HT produces two distinct responses in PFC pyramidal neurons, the 5-HT_1A_-mediated membrane hyperpolarization and the 5-HT_2_-mediated membrane depolarization [46]. Interestingly, the 5-HT-induced depolarization gradually shifts to a hyperpolarization commencing during the third postnatal week [45]. Electrical stimulation of the raphe nuclei elicits 5-HT_1A_-mediated inhibition and 5-HT_2A_-mediated excitation in PFC pyramidal neurons [47]. Moreover, 5-HT exerts a potent control on slow and gamma oscillations in PFC through 5-HT_1A_ and 5-HT_2A_ receptors, and induces distinct effects on the spiking of FS interneurons *in vivo* [26]. In this study, we found that the 5-HT_1A_-mediated decrease of intrinsic excitability is the predominant effect of 5-HT in PFC pyramidal neurons from young adult rats (∼4 wk), while the 5-HT_2_-mediated increase of intrinsic excitability is the predominant effect of 5-HT in PFC FS interneurons. Since blocking synaptic transmission did not alter the 5-HT regulation of AP firing, the opposing effects of 5-HT_1A_ and 5-HT_2_ on neuronal excitability could be attributable to their coupling to distinct ion channels including various K^+^, Ca^2+^, or cation channels [48]–[51].

Fluoxetine is an antidepressant drug whose therapeutic effect is considered to be through the inhibition of serotonin reuptake and the enhancement of serotonergic neurotransmission [36], [15]. Fluoxetine also has several other modulatory effects, such as inhibition of G protein-coupled receptors, blockade of monoamine oxidases and modulation of Ca^2+^ channels [52]–[54]. The effect of fluoxetine on PFC neuronal excitability has been largely unknown. In this study, we found that acute *in vitro* application of fluoxetine exerts a cell type-specific action in PFC, with a major impact on FS interneurons rather than pyramidal neurons. The smaller effect of fluoxetine on APs, compared to the effect of exogenously applied 5-HT, could be due to the limited level of endogenous 5-HT in PFC slices. However, chronic *in vivo* administration of fluoxetine also selectively alters the excitability of FS interneurons, confirming that FS interneurons are more sensitive to elevated endogenous 5-HT levels. It awaits to be investigated what determines the selective sensitivity to fluoxetine. Since the effect of exogenous application of 5-HT is largely occluded in FS interneurons from fluoxetine-treated animals, it suggests that 5-HT and fluoxetine converge onto a common set of membrane mechanisms to increase interneuron excitability.

## Materials and Methods

### Electrophysiology recording in slices

All experiments were carried out with the approval of State University of New York at Buffalo Animal Care Committee. Brain slices containing PFC from young adult male rats (∼4 weeks old) were prepared as described previously [55]. In brief, animals were anesthetized by inhaling 2-bromo-2-chloro-1,1,1-trifluoroethane (1 ml/100 g, Sigma St. Louis, MO) and decapitated; brains were quickly removed, iced, and then blocked for slicing. The blocked tissue was cut in 300–400 µm slices with a vibrating slicer (VT 1000 s, Leica, Nussloch, Germany) while bathed in a HEPES-buffered salt solution (in mM: 140 sodium isethionate, 2 KCl, 4 MgCl_2_, 0.1 CaCl_2_, 23 glucose, 15 HEPES, 1 kynurenic acid, pH 7.4, 300–305 mosM/liter). Slices were then incubated for 1–5 hr at room temperature (20–22°C) in a NaHCO_3_-buffered saline bubbled with 95% O_2_, 5% CO_2_ (in mM): 126 NaCl, 2.5 KCl, 2 CaCl_2_, 2 MgCl_2_, 26 NaHCO_3_, 1.25 NaH_2_PO_4_, 10 glucose, 1 pyruvic acid, 0.05 glutathione, 0.1 *N* ^G^-nitro-L-arginine, 1 kynurenic acid, pH = 7.4, 300–305 mosM. The slice was transferred to a perfusion chamber attached to the fixed-stage of an upright microscope (Olympus) and submerged in continuously flowing oxygenated artificial cerebrospinal fluid (ACSF). Neurons were visualized with a 40× water-immersion lens and illuminated with near infrared (IR) light, and the image was detected with an IR-sensitive CCD camera.

Whole-cell current-clamp recordings were performed using the similar approach as we described before [56]. Patch electrodes were filled with the internal solution (in mM): 60 K_2_SO_4_, 60 *N*-methyl-D-glucamine, 40 HEPES, 4 MgCl_2_, 0.5 EGTA, 12 phosphocreatine, 3 Na_2_ATP, 0.5 Na_3_GTP, 20 leupeptin, pH = 7.2–7.3, 265–270 mosM. Recordings were obtained with a DIGIDATA 1322A acquisition system and a Multiclamp 700A amplifier controlled by a computer running pClamp (Axon instruments, Foster City, CA). Action potentials were evoked by somatic injections of current pulses. The resting membrane potential was usually lower than −60 mV before being triggered to fire APs by the depolarizing pulses. Quantitative measurements were taken at 3–5 min after drug application. Numerical values were expressed as mean ± SEM. Statistical comparisons of drug effects were made using the student *t* test or ANOVA.

### Antidepressant treatment

Young male rats were administered intraperitoneally either with the antidepressant fluoxetine (10 mg/kg) or saline for 21 days (once daily) as we described before [31]. Experimental groups were matched such as a fluoxetine-treated rat and saline-treated control rat were sacrificed on the same day and tissues were processed in parallel.
